# Editorial: Population and clinical strategies for the prevention of type 2 diabetes: what’s new?

**DOI:** 10.3389/fendo.2023.1348018

**Published:** 2023-12-19

**Authors:** Maria Inês Schmidt, Pablo Aschner, Paula Andreghetto Bracco, William H. Herman

**Affiliations:** ^1^ Postgraduate Program in Epidemiology, School of Medicine, Universidade Federal do Rio Grande do Sul, Porto Alegre, Brazil; ^2^ Hospital de Clínicas de Porto Alegre, Porto Alegre, Brazil; ^3^ Javeriana University School of Medicine and San Ignacio University Hospital, Bogotá, Colombia; ^4^ Institution of Mathematics and Statistics, Universidade Federal do Rio Grande do Sul, Porto Alegre, Brazil; ^5^ Department of Internal Medicine, Division of Metabolism, Endocrinology, and Diabetes, University of Michigan, Ann Arbor, MI, United States; ^6^ Department of Epidemiology, School of Public Health, University of Michigan, Ann Arbor, MI, United States

**Keywords:** prediabetes, risk factors, screening, risk equations, intervention

Diabetes is a growing clinical and public health problem. The International Diabetes Federation estimates that 537 million adults 20-79 years of age are living with diabetes worldwide and that by 2045, the number will increase to 783 million (1). This timely and important Research Topic of Frontiers in Endocrinology addresses new population and clinical strategies for the prevention of type 2 diabetes. Investigators from around the world describe risk factors for prediabetes and type 2 diabetes, screening for risk of type 2 diabetes, and interventions to prevent type 2 diabetes. Investigators describe skipping breakfast as an independent risk factor associated with prediabetes among Japanese adolescents (Miyamura et al.) and depression and post-traumatic stress disorder following the great east Japan earthquake, tsunami, and nuclear disaster as independent risk factors for new-onset diabetes mellitus in Japan (Hirai et al.). Other studies document the value of waist-corrected body mass index in predicting incident diabetes (Wang et al.) and impaired circadian patterns of blood pressure regulation (including non-dipping blood pressure levels) as a risk factor for new-onset diabetes in hypertensive patients with obstructive sleep apnea in China (Luo et al.). Other studies demonstrate that both low and high triglyceride to high-density lipoprotein cholesterol ratios are associated with risk for incident type 2 diabetes among Japanese men with normoglycemia (Song et al.) and that serum levels of neprilysin, a membrane bound zinc-dependent type II metallopeptidase responsible for the breakdown of glucagon and glucagon-like peptide 1, are independently associated with both prevalent diabetes and the future risk of diabetes in Chinese adults (Hu et al.).

With respect to screening for risk of type 2 diabetes, an international group of investigators demonstrated that it was feasible to rapidly implement FINDRISC, a non-invasive screening tool for risk of type 2 diabetes based on age, body mass index, waist circumference, physical activity, daily intake of fruits and vegetables, history of hyperglycemia, history of antihypertensive drug treatment, and family history of type 2 diabetes, as part of a population-based screening campaign to detect people at risk for type 2 diabetes in 19 Latin America and Caribbean countries (Nieto-Martinez et al.). A second study based on a national survey of Brazilian adults demonstrated that most adults had good access to blood glucose testing and medical consultation (Santos et al.). Improvements in access were documented between 2013 and 2019 and in 2019, 77% reported having a glycemic test and 89% reported having access to a medical consultation in the past 2 years (Santos et al.). A third study, also from Brazil, demonstrated that risk equations that incorporate demographic, socioeconomic, lifestyle, clinical, and laboratory variables performed substantially better in predicting diabetes than fasting plasma glucose, 2-hour plasma glucose, and glycated hemoglobin used alone or in combination (Bracco et al.). Scores derived from multivariable equations that use continuously expressed clinical and laboratory variables detected more cases, identified fewer false positives, and consistently outperformed strategies based on categorical glycemic cut-offs (Bracco et al.).

With respect to interventions for diabetes prevention, a critical review of websites sponsored by business, government, and nonprofit organizations found that the top websites inadequately discussed dietary issues as causes, risk factors, and prevention strategies for type 2 diabetes (Crummett and Aslam). Websites are much more likely to discuss non-dietary lifestyle-associated risk factors such as physical activity and non-modifiable risk factors such as age (Crummett and Aslam). Based on the evidence that dietary interventions, with or without physical activity, can significantly decrease type 2 diabetes risk in both high-risk and general populations, the article concluded that diabetes websites should make a concerted effort to include more information about diet when discussing the causes, risk factors, and prevention of type 2 diabetes (Crummett and Aslam). Finally, in an article based on the report of the National Clinical Care Commission, a commission charged by the United States Congress to make recommendations to better leverage government policies and programs to more effectively prevent and control diabetes and its complications in the United States, the authors called for increased recognition of diabetes as a complex societal problem as well as a biomedical problem (Herman and Schillinger). They argued that the prevention and control of type 2 diabetes in the United States must begin with concrete population-level interventions to address social and environmental determinants of health including government policies and programs that address food and agriculture, education, housing, transportation, trade, commerce, and the environment (Herman and Schillinger).

Better definition of risk factors for type 2 diabetes, simpler and more efficient screening approaches to identify at-risk individuals, and both targeted and population level interventions are needed to prevent type 2 diabetes. We hope that this Research Topic of papers will provide you with insights about new opportunities and strategies to address the pandemic of type 2 diabetes.

## Author contributions

MS: Writing – review & editing. PA: Writing – review & editing. PB: Writing – review & editing. WH: Writing – original draft.
